# 558. Implementation and Outcomes of a COVID-19 Monoclonal Antibody Treatment Program in an Urban Safety-net Community Hospital

**DOI:** 10.1093/ofid/ofab466.756

**Published:** 2021-12-04

**Authors:** Alfredo J Mena Lora, Stephanie L Echeverria, Fischer Herald, James McSweeney, Harshil Gumasana, Rodrigo M Burgos, Scott Borgetti

**Affiliations:** 1 University of Illinois at Chicago, Chicago, IL; 2 Saint Anthony Hospital, Chicago, IL; 3 University of Illinois at Chicago College of Pharmacy, Chicago, IL

## Abstract

**Background:**

Neutralizing monoclonal antibodies (mAbs) bind to the receptor binding domain of the spike protein of SARS-CoV-2. In November 2020, several mAbs were issued an EUA by the FDA as single-dose intravenous (IV) infusions for treatment of mild to moderate COVID-19. mABs were allocated to local health facilities capable of administering infusions and managing side effects. Creating an outpatient infusion program during the COVID-19 winter surge can be logistically difficult. Our goal was to implement a mAb outpatient infusion program at an urban safety-net community hospital designed to serve communities most heavily impacted by COVID-19.

**Methods:**

The emergency department (ED) fast-track was repurposed for the mAb program with protocols from the infectious diseases physician and antimicrobial stewardship. Education materials with indications for mAbs were distributed in surrounding clinics serving our community. The program was available to all patients meeting criteria outlined in the protocol, 24/7, including but not limited to current ED patients and referrals from facilities in the vicinity.

**Results:**

Between December 1, 2020 and March 1, 2021, a total of 37 patients were treated: 51% male, 57% Hispanic or Latinx, 27% Black, and 95% (35) represented ZIP codes with high COVID-19 burden (Figure 1). Bamlanivimab was used for each instance and all infusions met criteria. Patient indications for mAb infusion are listed in Figure 2. Parenteral antibiotics were given to 10.8% and 35% received oral antibiotics upon discharge. At 30 days post-infusion, 8% (3) required hospitalization and there were no deaths.

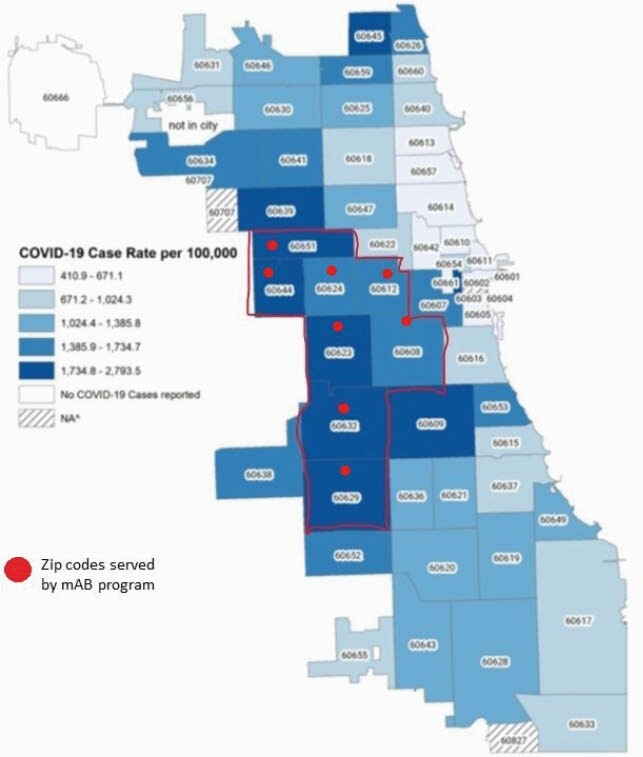

Zip codes with high COVID-19 disease burden served by our mAB infusion program

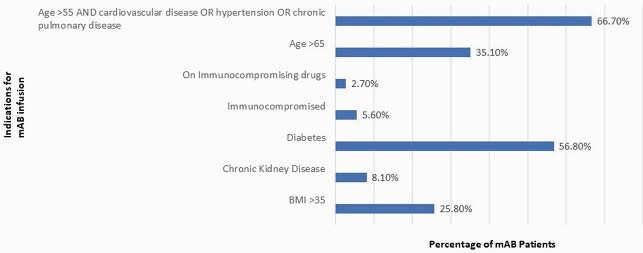

Distribution of patients who received mAB infusions by indication

**Conclusion:**

A mAb outpatient infusion program was successfully deployed in a safety-net community hospital. We believe strengths of the program included the flexible infusion hours and convenient referral site for patients and providers. Of importance, this program was able to provide services to minorities from ZIP codes most heavily impacted by COVID-19. Unfortunately, antibacterial use was common and may reflect broader unnecessary use in COVID-19 patients. Whilst mAb treatment was deemed appropriate in all instances via protocol inclusion criteria, antibacterial stewardship programs may need to expand to ED settings for COVID-19 management.

**Disclosures:**

**All Authors**: No reported disclosures

